# Supplementary material for: Intralumenal docking of connexin 36 channels in the ER isolates mistrafficked protein
Source: J Biol Chem. 2023 Sep 22;299(11):105282. doi: 10.1016/j.jbc.2023.105282 (PMC10637963; doi:10.1016/j.jbc.2023.105282)

**Supplementary information for**

**Intralumenal docking of Cx36 channels in the ER isolates**

**mis-trafficked protein**

Authors: Tetenborg et al., 2023

*Corresponding author: Email: [stetenbo@Central.UH.EDU](mailto:stetenbo@Central.UH.EDU) (S.T) and [jobrien3@Central.UH.EDU](mailto:jobrien3@Central.UH.EDU) (J.OB.)


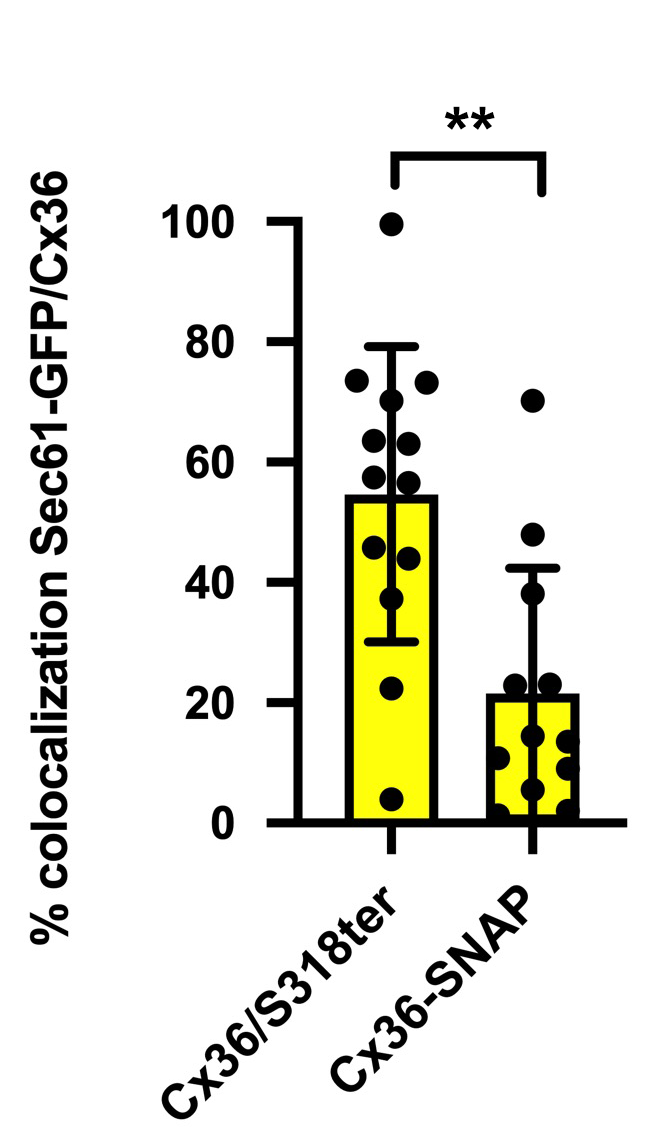


**Supplementary figure 1: Degree of colocalization between different Cx36 variants and Sec61-GFP.** Colocalization was quantified as the relative area of the Cx36 channel labeled by Sec61-GFP. Data show SD. T-test. P<0.001

**Supplementary figure 2: Double labeling of Cx36-SNAP and ERGIC53 or Rab11. (A)** Cx36-SNAP does not colocalize with ERGIC53**. (B**) Cx36 shows little to no association with the endosomal marker rab11. Occasionally, Rab11 positive puncta are localized in the center of a whorl. Scale:10 micron.


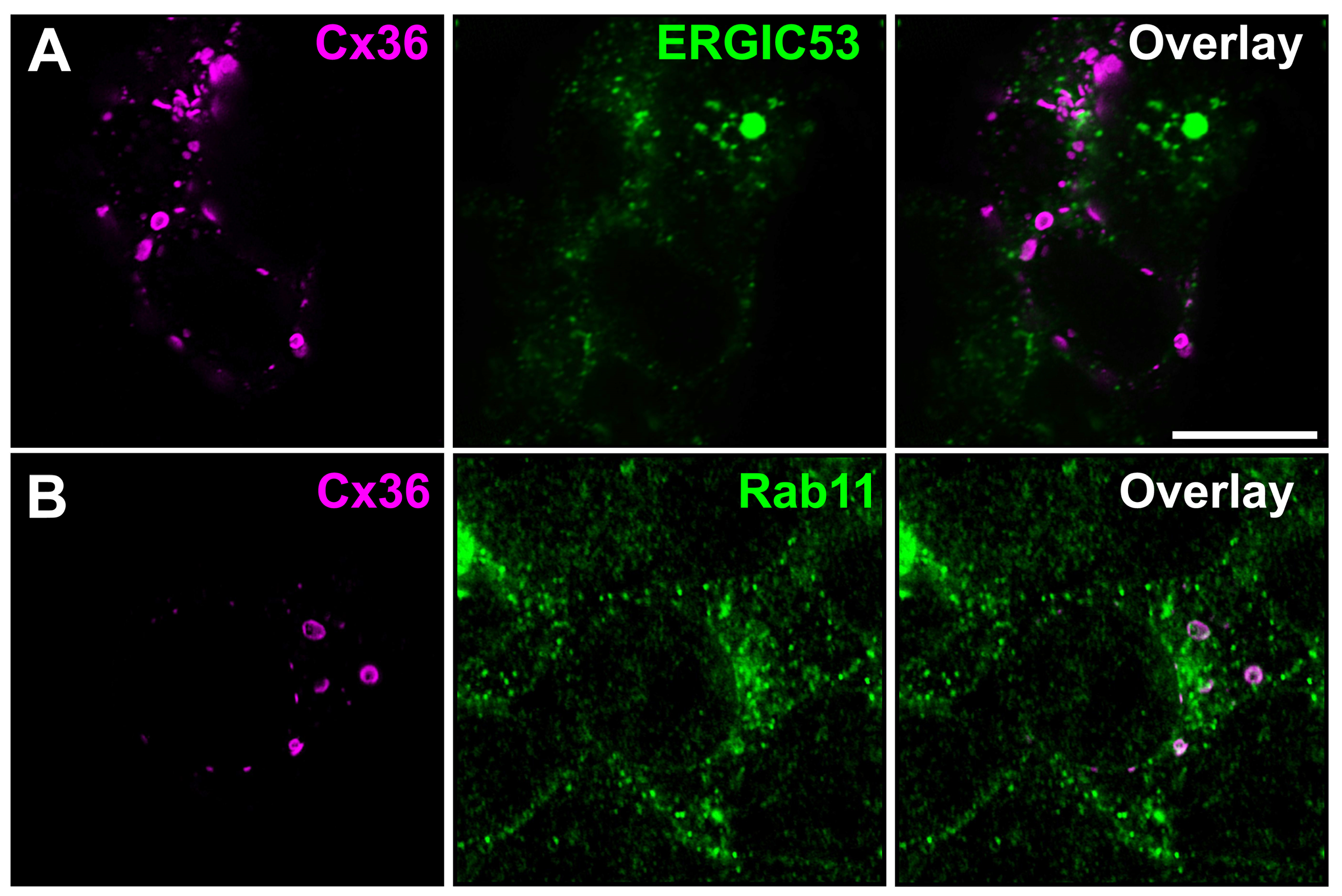

Supplement: Supplemental Figs. S1 and S2 [file mmc1.docx]
